# Point-of-Care Tests for Bladder Cancer: The Influencing Role of Hematuria

**DOI:** 10.1155/2011/937561

**Published:** 2011-11-22

**Authors:** Joerg Hennenlotter, Severine Huber, Tilman Todenhöfer, Ursula Kuehs, David Schilling, Stefan Aufderklamm, Georgios Gakis, Christian Schwentner, Arnulf Stenzl

**Affiliations:** Department of Urology, Eberhard Karls University Tuebingen, Hoppe Seyler Stra*β*e 3, 72076 Tuebingen, Germany

## Abstract

*Introduction*. Several point-of-care tests (POCT) are available for the diagnosis of bladder cancer (BC). We evaluate the impact of HU (hematuria) on performance of POCTs. *Materials and Methods*. Urine from 10 donors was diluted with blood from 0.5 to 0.00625%. BladderCheck^R^, BTAstat^R^, BCM^R^, and BTA^R^ tests were applied. Tests were additionally conducted in 54 patients with HU. HU was stratified according to the amount of erythrocytes (RBC)/**μ**L using two systems: (1) no HU; mild microscopic HU; severe microscopic HU; gross HU; (2) I <25 RBCs; <250 II; ≥250 III. Results were compared to HU status and histopathology. *Results*. Gross HU became evident between 2090 RBCs/**μ**L and 1065/**μ**L. Addition of blood led to default tests in all 4: BladderCheck^R^ 0.25%; BCM 0.025%, BioNexia 0.00625%, and BTAstat <0.00625%. Rates of false positives for BladderCheck, BTAstat, BCM, and BioNexia were 5.9, 11.8, 0, and 1.8% without HU and 0, 66.7, 44.4, and 66.7% with HU. BTAstat, BCM, and BioNexia were independently influenced by HU (*P* < 0.0002). *Conclusions*. NMP22-BladderCheck was most resistant to blood. The diagnostic yield of all others was significantly influenced by HU. A well-defined HU grading helps to define limits of HU for a reliable interpretation of BC-POCTs.

## 1. Introduction

Bladder cancer (BC) presents as a malignancy with increasing incidence, a significant impact on quality of life, and the highest lifetime treatment costs per patient [1]. Early detection to prevent progressive disease remains the challenge of the disease. Since specific symptoms are lacking and most patients present with unspecific hematuria (HU), invasive measures are still needed for ultimate diagnosis. Hence, cystoscopy is still the gold standard in the diagnostic workup of BC [2]. Noninvasive tests are restricted to urine markers. Beside urine cytology, molecular markers evolved within recent years and play an emerging role in diagnosis and monitoring of BC [3]. However, the diagnostic accuracy of cytology and of molecular markers remains unsatisfactory. Due to insufficient discriminatory power, urine markers are still not able to replace cystoscopy [4]. Recently, office-based point of care test (POCT) systems became available [5]. They are particularly useful for office physicians without direct access to cost-intensive laboratory equipment.

Several sources for limited diagnostic yield, mainly for limited specificity, have been identified. Whereas urinary tract infection, mechanical stress due to instrumented urinary sampling and others could be determined as influencing factors for several tests [6–8], HU has not been extensively studied yet [6, 9]. Even though a high percentage of patients presents with HU, its drawbacks on POCTs are poorly defined. HU, however, covers a wide spectrum reaching from single RBCs to apparent gross HU. Even though others have reported on the interaction of blood and urine-based tests for BC, there are no clear-cut reports available about the impact of different grades of HU on such tests [10–12]. Furthermore, evidence is even more limited when focusing on the susceptibility of POCTs to HU.

Aim of the study was to investigate the influence of well-defined grades of HU on four commercially available POCTs in an experimental setting as well as in patients suspected to have BC.

## 2. Materials and Methods

### 2.1. In Vitro Study

**Figure 1:**
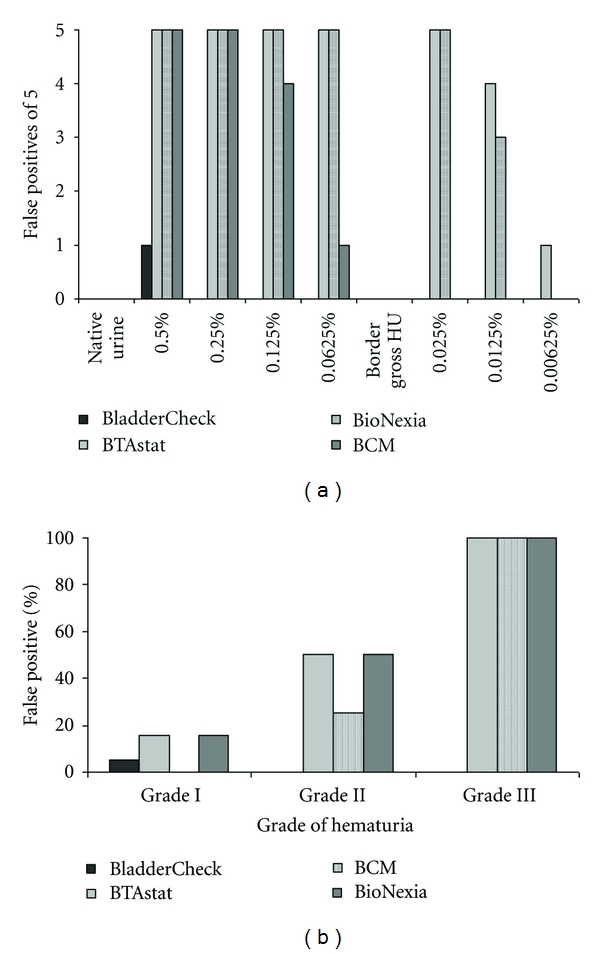
(a) False positive results of POCTs out of five patients per setting in different dilutions. HU: hematuria. (b) Rates of false positive test results and grades of hematuria in BC patients.

Ten subjects who presented for routine checkup with no evidence of BC or other disease were included. Median age was 44 (range 21–55) years, 6 were males, and 4 were females. Midstream urine of at least 100 mL was collected in all. Cytology, dipstick, and microscopy of the investigated urine specimen were inconspicuous. Patients did not have signs of uro-/nephrolithiasis, urinary tract infection, or cystitis. The study received approval by the local IRB (no. 400/2009A). Autologous blood was drawn and stored in an EDTA tube. Subsequently, a hemogram was performed. By addition of 0.2 mL whole blood to 19.8 mL urine, a 1.0% v/v dilution was generated, and, by performing serial dilutions, additional concentrations were generated as shown in Figure 1. To allow for potential interactions between urine and blood and to generate grades of HU, urines were incubated for five minutes after blood addition. In every dilution, a dipstick test was performed and RBCs were counted using a Neubauer-improved counting chamber. NMP22 BladderCheck^R^ (Matritech, Freiburg, FRG), BTAstat^R^ (Polymedco, Cortlandt Manor, NY, USA), BioNexia-BTA^R^ (BioNexia, Göttingen, Germany), and BCM^R^ (Ulti med GmbH, Ahrensberg, Germany) were performed simultaneously according to the manufacturer's recommendations within 30 min after urine sampling.

### 2.2. Clinical Study

54 consecutive patients were enrolled between 06/2009 and 11/2009. Patients were either suspicious of having BC or had histologically proven muscle-invasive BC. The first group subsequently underwent cystoscopy and the second group had radical cystectomy. 54 patients before cystoscopy (*n* = 48) or cystectomy (*n* = 6) were investigated. Median age was 68.5 (range 42–90) years, 42 were males, and 12 were females. No patient had mechanical manipulation of the urethra or bladder within the last seven days before specimen harvesting. Patients with a recent history of urolithiasis, urinary tract infection, cystitis, or previous Mitomycin/BCG installation were excluded. Dipstick and urine microscopy were performed, and the presence of HU was documented.

HU was subclassified by dipstick, urine microscopy, and visual assessment: no RBCs neither in dipstick nor in microscopy = no HU; RBCs 10–250/*μ*L in dipstick and microscopy = mild micro-HU; >250/*μ*L RBCs in dipstick and microscopy = severe micro-HU; visible blood colouring = gross HU. Since initial results had already shown considerable differences already in low-grade HU, HU was further subclassified in a second step: RBCs ≤25 (including 0) = Grade I, <25 RBCs <250 = Grade II, and RBCs ≥250 including gross HU = Grade III.

The amount of false positive tests was comparatively investigated for all POCTs. For the patients' cohort, the relation of HU and the presence or absence of BC were analyzed by contingency tables. False positive results were further associated with the predefined grades of HU. Multivariate analysis was done to assess the independent impact of HU on the diagnosis of BC.

## 3. Results

### 3.1. In Vitro Results

The serial dilutions (0.5%-0.25%-0.125%-0.0625%-0.0025%-0.00125%-0.000625%) of the harvested urine yielded the following values: ***21.200*** RBCs/*μ*L = **0.5%**; ***10.730*** RBCs/*μ*L = **0.25%**; ***4.080*** RBCs/*μ*L = **0.125%**; ***2.090*** RBCs/*μ*L = **0.0625%**; ***1.065*** RBCs/*μ*L = **0.0025%**; ***326*** RBCs/*μ*L = **0.00125%**; ***168*** RBCs/*μ*L = **0.000625%**. Dipstick RBC counts correlated well to the microscopy. The threshold level from mild to severe microscopic HU was found to be between a dilution of 0.0125% and 0.00625%. At 0.0625% (corresponding to 2090 RBCs/*μ*L), but not at 0.025% (1065/*μ*L), gross HU was clearly notable. The addition of blood led to default tests in all 4 POCTs. However, the level of dilution was different: NMP22-BladderCheck^R^ was the most stable test system at 0.25% (10.730/*μ*L) followed by BCM, BioNexia, and BTAstat. BCM was altered at 0.025% (1065/*μ*L), BioNexia at 0.00625% (168/*μ*L), and BTAstat at <0.00625% (<168/*μ*L) for (Figure 1(a)).

### 3.2. Patients' Results

Of the patients before cystoscopy, 8 presented with a history of BC during followup. After TUR or cystectomy, 28/54 patients (51.9%) were found to have BC: 17 × Ta, 4 × T1, 5 × T ≥ 2, and 2 × CIS. G1 tumors were found in 7, G2 in 13, and G3 in 6 patients.

At the time of enrolment, 32/54 (59.3%) of the patients had HU. According to the predefined criteria, 20 subjects had mild microscopic HU, 8 had severe microscopic HU, and 4 had gross HU. When the adjusted grading for HU was used, 31 patients had grade I (including no HU) HU, 6 grade II HU, and 17 grade III (including gross HU). Contingency tables of HU and presence of BC are shown in Table 1. Results from contingency analysis of graduated HU and false positive tests are shown in Figure 1(a). In the group of patients with proven BC, 12 had grade I HU (42.9%), 2 grade II HU (7.1%), and 14 grade III (50.0%, Table 1).

17/26 (65.4%) patients who had no BC presented without HU. Of those, 1 (5.9%), 2 (11.8%), 0 (0.0%), and 2 (11.8%) patients had false positive results of BladderCheck, BTAstat, BCM, and BioNexia, respectively. False positive test results were seen in 9/26 patients who had no BC but HU: 0 (0%) for BladderCheck, 6 (66.7%) for BTAstat, 4 (44.4%) for BCM, and 6 (66.7%) for BioNexia. 2/26 patients presented without BC but had severe microscopic HU or gross HU, and false positive results were seen in 0 (0%) for BladderCheck and 2 (100.0%) patients for BTAstat, BCM, and BioNexia, respectively. One patient presenting without BC but gross HU revealed a negative BladderCheck, whereas BTA, BCM, and BioNexia were positive.

### 3.3. Classification of HU according to Grade

Patients presenting without BC were found to have various degrees of HU: 19 × grade 1 (73.1% of all 26 patients without BC), 4 × grade 2 (15.4%), and 3 × grade 3 HU (11.5%, Table 1). False positive results were documented for BladderCheck, BTAstat, BCM, and BioNexia (Figure 1(a)). At grade 1, HU in 1 (5.3%), 3 (15.8%), 0 (0%), and 3 (15.8%) of 19 patients; at grade 2, HU in 0 (0%), 2 (50.0%), 1 (25.0%), and 2 (50.0%) of 4 patients; at grade 3, HU in 0 (0%), 3 (100%), 3 (100.0%), and 3 (100.0%) of 3 patients, respectively, (Figure 1(a)).

In multivariate analysis, HU was an independent influencing variable for the results of BTAstat, BCM, and BioNexia (*P* = 0.0002, <0.0001, and <0.0001). The NMP22-BladderCheck test was not significantly influenced by HU (*P* = 0.77).

## 4. Discussion

Molecular urine tests are promising biomarkers for BC. However, they are still not well established in daily clinical routine and in the standard diagnostic workup of BC [2]. In contrast to other molecular tests that require elaborate molecular methodology (e.g., FISH, immunocytology, or ELISA), new POCT systems provide the opportunity to be applied by office physicians without direct access to expensive laboratory equipment. However, for being broadly used, adequate test specificity and sensitivity is at least as important as the feasibility.

Several studies have been published comparing accuracy of various POCTs. Sensitivity and specificity are widely varying within different publications [3, 5, 13–15]. These variations may be explained by inherent influencing variables such as infection or hematuria. Data on such interferences are scarce, and experimental data are even less common. Hence, we designed the present study to investigate the impact of such a variable on 4 of the most commonly used POCTs for BC detection. An experimental model for different grades of HU was constructed allowing for accurate assessment of the influence of the RBC count on the test system. Moreover, a threshold for default could be determined for all POCTs. Since all 4 POCTs adhere to different principles, it is not very surprising that there were clear differences between the investigated test systems. The BladderCheck showed a positive result only in one subject at a dilution of 0.5% and was then negative for all following dilutions. All other tests were positive throughout 0.25%, and the BCM test showed one negative result out of five at 0.125% and became totally negative at 0.025%. Gross HU leads to false positive results in BTAstat and Bionexia, whereas the BladderCheck showed no false positive results under such circumstances. The BCM test was indeterminate being only partly susceptible to HU. In microscopic HU conditions, only BladderCheck and BCM remained constantly negative whereas BTAstat and BioNexia were not resistant to even very small amounts of blood in the urine.

Gross HU is a clear indication to perform cystoscopy and upper tract imaging. However, there is no well-defined and widely adopted algorithm for the evaluation of microscopic HU. As many practicing physicians are hesitant to recommend cystoscopy to such patients due to its invasiveness, POC tests are of particular interest serving as an additional decision tool.

The results of the in vitro setting were further validated in a consecutive cohort of patients. In the HU group, no BladderCheck was false positive, the BCM showed lower susceptibility to blood than BTAstat and BioNexia. In contrast to the in vitro setting, even severe microscopic HU resulted in a 100% false positivity of the BCM test. When HU was graded from I to III, the latter test showed improved performance with a rate of only 50% false positives below grade III.

NMP22 was reported to be overexpressed by malignant urothelial cells and released into the urine by apoptotic cells [16]. Therefore, other nonmalignant conditions producing apoptotic cells may provide NMP22 in the urine [3]. As RBCs do not have a nucleus, they cannot increase NMP22 directly in HU samples. Possible explanations for the impact of RBC might be membrane factors causing a cross-reaction or related serum proteins [11]. The remaining investigated assays detect the human complement factor H and related protein (the Bladder Tumour-associated Antigen) which is reported to be a marker for BC [17]. Blood-bound H-related protein could be responsible for false positive assays [12].

Experimental spiking of blood into urine was already introduced by other groups. In contrast to our study, which focuses on the performance of POCTs, Atsü et al. investigated the quantitative NMP22-ELISA [11]. They conducted serial dilutions and found artificially increased results above the ELISA cut-off level between 0.2 and 0.02% blood. Compared to the present results, their report indicates that the quantitative test might shift positive earlier than the POC test. However, they refered their dilutions to high field counts and did not determine exact RBC concentrations.

Yokoyama et al. [18] provided first data on the BladderCheck: they reported false positive results above 10^5^ RBCs/*μ*L. Both studies confirm that the BladderCheck is resistant to microscopic HU being an extremely frequent condition in affected patients. Oge et al. [12] used relatively high concentrations of blood admixtures and found a 20% false positivity of the BTAstat at a dilution of 1 : 2000 (0.05%); however, this dilution might be close to microscopic HU. The results of both were confirmed by our results.

Schwentner et al. presented data about the influence of HU on results of urine tests in a cohort of more than 2000 patients. They confirmed a clear influence of HU on the performance of the NMP22-ELISA. Moreover, a clear influence of HU on immunocytology and even cytology was reported [19]. The fact that in our study test systems did not show uniform results at one dilution threshold (either all positive or all negative) also indicates additional factors in urine that impact on the test outcome.

Grading of HU is increasingly addressed by studies evaluating urine tests. Pesch et al. suggested in a prospective study a semiquantitative recording of urine RBCs to more accurately interpret positive NMP22 (ELISA) results [10]. The dilution experiments performed in the present work allow for quantifying HU into distinct subgroups: the upper limit of the dipstick is 250 RBCs/*μ*L. However, gross HU was not visible before 2000/*μ*L. All tests have been shown to perform clearly better below a 250 RBC dipstick result.

The limitation of our study is a relatively low number of patients. Moreover, as our study only evaluated the impact of HU on false positive results, we cannot comment on the influence of sensitivity on the rate of false positivity. NMP22 is a strong, independent predictor of bladder cancer. Integration of NMP22 into clinical decision making helps avoid unnecessary cystoscopies [20].

This is the first study comparing the impact of HU on the performance of 4 POC urine tests for BC in an experimental setting. We found a correlation between the false positive rate of BTAstat, BMC, and BioNexia tests and urinary blood burden. The diagnostic properties of BladderCheck were not influenced by HU. Our findings may contribute to a more comprehensive understanding of HU grading providing a clinical classification of HU. A well-defined HU grading may help to define appropriate limits of HU for a reliable application and interpretation of BC-POCTs in the office setting.

## Figures and Tables

**Table 1:** Contingency table for the presence of bladder cancer and graded hematuria.

Counts Total % Column % Line %	No bladder cancer	Bladder cancer	
No hematuria	**17** 31,48 65,38 77,27	**5** 9,26 17,86 22,73	**22** 40,74
Hematuria	**9** 16,67 34,62 28,13	**23** 42,59 82,14 71,88	**32** 59,26

Grades of hematuria			
Grade I	**19** ** 35,19** ** 73,08** ** 61,29**	**12** ** 22,22** ** 42,86** ** 38,71**	**31** ** 57,41**
Grade II	**4** ** 7,41** ** 15,38** ** 66,67**	**2** ** 3,70** ** 7,14** ** 33,33**	**6** ** 11,11**
Grade III	**3** ** 5,56** ** 11,54** ** 17,65**	**14** ** 25,93** ** 50,00** ** 82,35**	**17** ** 31,48**

Total	**26** ** 48,15**	**28** ** 51,85**	**54**
